# Effects of Social Media Narratives on Affective and Behavioral Responses to Menopause Content: Randomized Online Experimental Study

**DOI:** 10.2196/85788

**Published:** 2026-07-17

**Authors:** Alison K Osborne, Richard D Brown, Elizabeth Sillence

**Affiliations:** 1 Northumbria University Newcastle United Kingdom

**Keywords:** menopause, social media, narrative framing, women’s health, digital health

## Abstract

**Background:**

Social media is an increasingly prominent channel for communicating menopause information and experiences, yet the affective and behavioral consequences of different narrative framings remain unclear.

**Objective:**

We examined how distress, normalizing, and transformative narratives influenced women’s immediate responses to menopause content online, drawing on established narrative framings of menopause as normality, distress, and transformation.

**Methods:**

In an online experiment, UK women aged 40 to 83 years who were perimenopausal or postmenopausal were recruited via Prolific, a widely used online recruitment platform for behavioral and social science research. A total of 737 women were randomly assigned to view 4 anonymized and standardized social media posts from a pool of 12 reflecting 1 of 3 narratives: *normal* (n=248, 33.6%), *distress* (n=241, 32.7%), or *transformative* (n=248, 33.6%). Participants then reported affective reactions, expected behavioral responses, and perceptions of the posts using 5-point ordered response scales. Ordinal logistic regression models tested demographic predictors and condition effects controlling for demographic factors.

**Results:**

Participants who viewed distress-framed posts reported greater levels of worry (β=.910; *P*<.001), confusion (β=.818; *P*<.001), and anxiety (β=.817; *P*<.001) and lower levels of reassurance (β=−.970; *P*<.001), optimism (β=−.708; *P*<.001), and empowerment (β=−.540; *P*<.001). Distress framing also increased perceived knowledge of menopause (β=.564; *P*<.001) despite participants feeling more negatively toward the posts. Neither distress nor transformative narratives influenced expected behavioral intentions to like, share, save, comment on, search for, or discuss social media posts compared with normalizing narratives. Postmenopausal status (β=−.630; *P*<.001) and older age (β=−.492; *P*<.001) were independently associated with less worry and anxiety. Participants rated distress (β=−.806; *P*<.001) and transformative posts (β=−.968; *P*<.001) as less representative of health professionals than normalizing posts; transformative posts were also judged to be less representative of newspapers or television (β=−.687; *P*<.001).

**Conclusions:**

Narrative framing shaped immediate affect but not intended engagement with menopause content. Because this study assessed short-term responses to controlled, standardized posts, future research should examine whether these effects persist over time and how they operate in more ecologically valid social media environments. As public discussion expands, diverse, balanced narratives may help reduce stigma and temper the disproportionate salience of negative framing. This study advances understanding of how narrative framing shapes responses to health content online.

## Introduction

Menopause usually occurs between the ages of 44 and 55 years, with approximately 1 in 100 women experiencing menopause prior to the age of 40 years [1]. The age at which women experience menopause is highly variable, and menopause can be induced as a result of surgery, serious illness, or medication [2,3]. Consequently, in an aging population, women spend more than a third of their life being perimenopausal, menopausal, or postmenopausal [4]. Existing information available about the menopause transition can be conflicting regarding both experiences and symptomology. Over the years, messaging regarding menopause has often been framed negatively by the media and subject to misinformation and stigma [5]. Earlier discourses on menopause have proposed links to hysteria, mental health issues, and female hormones [6], and more recently, the focus has moved toward menopause as a medical problem to be solved [7]. Several studies have indicated poor knowledge and awareness of the menopause transition in women [8,9]. Due to this lack of awareness, women are less likely to seek or receive the medical assistance they require [10] and have often only proactively sought information about menopause through self-directed learning after their 30s [11].

Many people use the internet as their first place for seeking health information over consulting friends, family members, or health professionals [12]. New technologies have changed the way in which we communicate, and there is a growing abundance of online health information; however, online health resources are often poorly regulated [13]. In particular, social media has become a key tool for health communication as it allows users to share information across geographical boundaries and create online communities [14]. Health information on social media comes from a wide range of sources, including celebrities, influencers, health professionals, charities, advocacy groups, and the public [15-17]. Consequently, individuals can engage with diverse perspectives on menopause, which may influence their perceptions and emotional responses. There is a growing interest in how social media affects public perceptions of health, with an increase in the use of social media for health communication [12,18]. Engaging with social media can empower individuals by improving access to information about menopause and increasing perceptions of control and participation in important medical decisions [19,20]. However, the extent to which narratives online affect individuals’ perceptions of menopause is not wholly understood. Research has shown that women have diverse emotional responses to menopause, with many expressing a range of views from positive and accepting to negative and fearful [21]. These varied emotional responses highlight the importance of exploring how different social media narratives can impact women’s views, offering insights into how these may either challenge or reinforce existing perceptions. We focused on perimenopausal and postmenopausal women because these groups were most likely to have current or lived experience of the menopause transition. This enabled us to examine responses to menopause-related social media narratives among women for whom the content was personally relevant while also allowing for comparison between those currently experiencing the transition and those reflecting on it after menopause. De Salis et al [22] investigated experiences and perceptions of menopause among UK mothers, identifying three interdependent narratives: (1) *normality*—menopause as a normal, biological process distinct from the self, identity, and social transitions; (2) *distress*—menopause as a struggle, provoking and expressing distress, and a time of identity loss and social upheaval; and (3) *transformation*—menopause as a time of liberation and positive change. In this paper, we use the term “social media narratives” to refer to recurring ways of framing menopause in social media content, including the meanings, emotional tone, and implied interpretations attached to the menopause transition. These narrative types provide a useful framework for understanding how different social media narratives may impact individual perceptions of menopause.

Given the increasing role of social media in shaping public discourse, understanding how different narratives influence perceptions of menopause is crucial for effective public health communication. A recent literature review highlighted the paucity of research on the use of social media for menopause messaging [23]. Consequently, this study aimed to understand how women respond to different social media narratives on menopause. The objectives of this study were as follows:

1. To assess the role of individual characteristics in shaping responses to different social media narratives about menopause
2. To examine the impact of different narratives on affective responses to menopause posts
3. To examine the impact of different narratives on behavioral responses to menopause posts
4. To evaluate women’s perceptions of different narratives and how representative they believe these are of views expressed by social media, traditional media, and health professionals

## Methods

### Design

This study used a between-subject online experimental design with 3 conditions. Each condition presented participants with a distinct set of 4 social media posts representing different perspectives on menopause. After viewing the content, all participants answered the same set of questions assessing participants’ affective, behavioral, and perceptual responses to the presented posts.

### Participants

Participants were recruited through Prolific, an online platform for study recruitment. Prolific was chosen because it is a widely used platform for recruiting online survey participants in behavioral and social science research, particularly in UK and US samples. Previous comparisons of online recruitment platforms have found Prolific to provide relatively high-quality data, including stronger performance on attention, comprehension, honesty, reliability, and internal consistency checks than several alternative platforms [24,25].

Eligibility criteria were specified through Prolific prescreening and study screening questions. Participants were eligible if they were female UK residents aged 40 years or older and self-reported being either perimenopausal or postmenopausal based on participants self-selecting the stage that best described them currently: “Perimenopause—experiencing symptoms such as hot flushes, brain fog, joint pain, changes in periods,” “Post menopause—not had a period for 1 year or more,” or “Not going through the menopause,” with only those selecting perimenopause or postmenopause being eligible to take part.

A sample of 749 participants completed the survey, of whom 4 (0.5%) were excluded from the analysis for reporting more than 20 years of post-16 education, suggesting a likely misunderstanding of the question. An additional 1.1% (8/749) were excluded for completing the survey in under 2.5 minutes, which was deemed by the research team to be insufficient time to adequately engage with and process the survey content. The final sample of 737 participants was randomly assigned to 1 of 3 experimental conditions: *normal* (n=248, 33.6%), *distress* (n=241, 32.7%), and *transformative* (n=248, 33.6%) narrative framing. The recruitment target was set pragmatically to obtain approximately 250 participants per experimental condition, providing a large and balanced sample for comparing responses across the 3 narrative conditions. This conservatively exceeded methodological guidance suggesting that between-subject psychological experiments often require at least approximately 200 participants per condition to achieve adequate power for plausible effect sizes [26]. Participants were compensated for taking part in the study in line with fair pay guidelines from Prolific and Northumbria University. Participants were aged between 40 and 83 years (mean 52.992, SD 8.336 years). Of these participants, 52.2% (385/737) reported being perimenopausal, and 47.8% (352/737) reported being postmenopausal.

### Participant Experience

**Figure 1:**
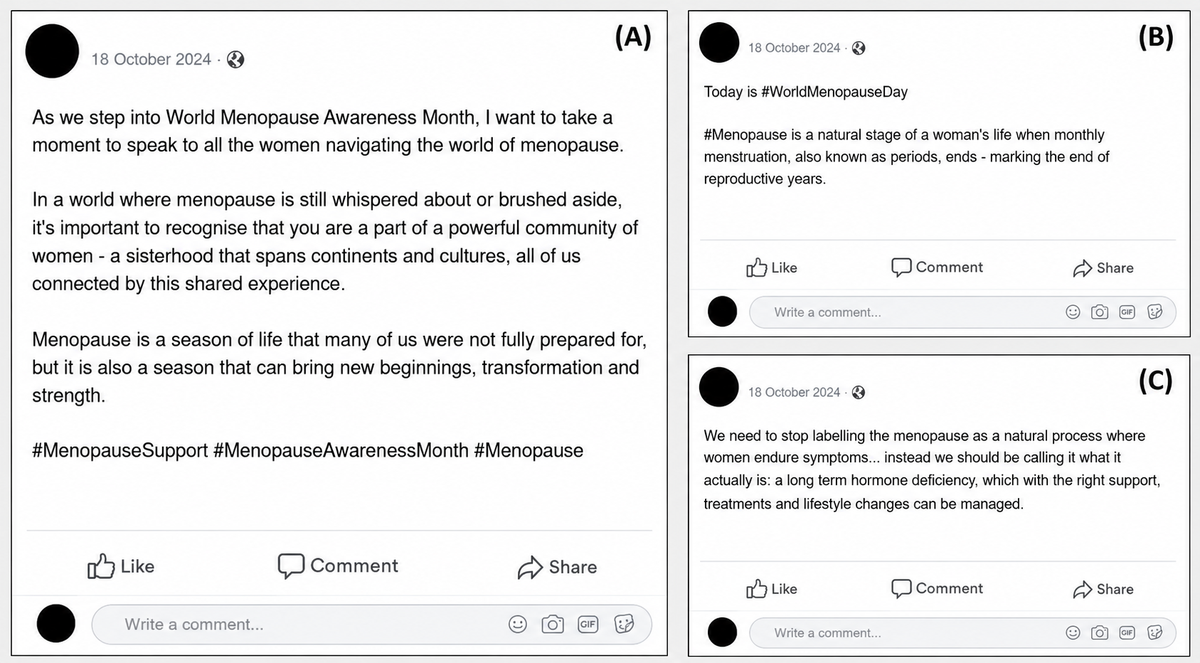
Examples of anonymized and standardized menopause-related social media posts used as stimuli in the (A) transformative, (B) normal, and (C) distress narrative conditions.

Following screening, participants were presented with an online Qualtrics questionnaire (Qualtrics International Inc). Participants first answered questions about their age, number of years in post-16 education, perceived knowledge of menopause, hormone replacement therapy (HRT) history, current feelings about menopause, and social media use. Participants were then randomly assigned to 1 of 3 conditions. These 3 conditions mapped onto the interdependent narratives of menopause identified by de Salis et al [22]: *normal*, *distress*, and *transformative*. For each condition, participants were presented with 4 social media posts that reflected the condition to which they were assigned, resulting in 12 posts in total: 4 normal, 4 distress, and 4 transformative posts. Posts were copied from X (formerly known as Twitter), Instagram, and Facebook posts for World Menopause Day 2024. All posts were publicly available at the time of creating the experiment materials but were anonymized, redacted, and standardized to remove any potential platform or content creator effects. Posts were initially coded into the normal, distress, and transformative narrative categories by 2 members of the research team based on their fit with the 3 menopause narratives identified by de Salis et al [22]. The categorization was then sense checked with a small group of perimenopausal and postmenopausal women (n=6) who completed a think-aloud exercise focused on the posts’ content, style, and language and how the posts made them feel about menopause. Participants were then asked to group the posts, providing an additional check on the narrative categorization used in the experiment. To ensure that participants had sufficient time to consider the posts presented as experimental stimuli, a 60-second timer prevented participants from progressing the survey. Participants were then asked to identify which message type (*normal*, *distressing*, or *transformative*) they believed the posts represented. Figure 1 shows examples of posts provided for each of the 3 conditions.

After being presented with the social media posts about menopause, participants from each experimental condition were asked the same series of questions. The questions were designed by the research team to capture immediate affective, behavioral, and perceptual responses to the posts. Items were developed to reflect the study aims and the anticipated ways in which menopause-related social media narratives might influence participants’ feelings, engagement intentions, and judgments of the posts. Participants responded using 5-point Likert-type scales. Affective response items asked participants the extent to which they agreed with statements such as “I feel worried about menopause,” “I feel reassured about menopause,” “I feel confused about menopause,” and “I feel optimistic about menopause.” Behavioral response items asked how likely participants were to take actions such as “‘like’ the posts,” “share the posts,” “save/bookmark/screenshot the posts,” “comment on the posts,” “search for other similar posts and messages,” or discussing the posts with “friends and family” or “a health professional.” Perceptual items asked participants the extent to which they agreed with statements such as “I liked the posts,” “I found the posts credible,” and “I trust the posts” and whether the posts were representative of menopause-related views on social media, in traditional media, and among health professionals.

### Ethical Considerations

This study was granted ethics approval through Northumbria University’s Ethics Online System (project number 9647). Participants were presented with an online participant information sheet at the start of the Qualtrics survey, which explained the purpose of the study, what participation would involve, the voluntary nature of participation, their right to withdraw before submitting their responses, how their anonymous data would be used, and researcher contact details. Participants were required to confirm informed consent online before proceeding to the study questions. All participant data were collected anonymously, and participants could not be identified from the data. Participants were reimbursed for their participation through Prolific at a rate of £1.50 (£1=US $1.32 as of June 29, 2026) in line with Northumbria University ethical guidelines for the fair payment of research participants.

### Statistical Analysis

All statistical analyses were performed using R (R Foundation for Statistical Computing) [27]. The following packages were used for data processing, analysis, and visualization: *apaTables* [28], *ggplot2* [29], *psych* [30], and *tidyverse* [31]. Our full R script is available on the Open Science Framework [32]. Descriptive statistics were used to summarize participant characteristics, HRT experience, perceived menopause knowledge, and social media engagement. Frequencies and percentages are reported for categorical variables, and means, SDs, and ranges are reported for continuous variables. Ordinal logistic regression was used for statistical inference because the outcome variables were measured using ordered 5-point Likert-type response scales. First, ordinal logistic regression models were used to investigate whether individual characteristics predicted affective and expected behavioral responses to menopause posts. Second, ordinal logistic regression models examined the impact of experimental condition on affective, behavioral, and perceptual responses to the posts using the normal narrative condition as the reference category. Models controlled for age, years of education, menopause status, and frequency of social media use where relevant. To mitigate the multiple comparison problem, a statistical significance threshold of a *P* value of less than .01 was adopted.

## Results

### Overview

Overall, most participants (519/737, 70.4%) reported never having undergone HRT, and most (593/737, 80.5%) felt that they were moderately, very, or extremely knowledgeable about menopause. Most participants (615/737, 83.4%) reported at least daily engagement with social media. A breakdown of participant levels of experience of HRT, knowledge of menopause, and social media engagement is presented in Table 1.

**Table 1:** Hormone replacement therapy (HRT) experience, perceived menopause knowledge, and social media engagement among UK perimenopausal and postmenopausal women by experimental condition (N=737).

		Normal framing condition (n=248), n (%)	Distress framing condition (n=241), n (%)	Transformative framing condition (n=248), n (%)	Total, n (%)
**Experience of HRT**					
	Currently undergoing HRT	45 (18.1)	38 (15.8)	54 (21.8)	137 (18.6)
	Previously undergone HRT	25 (10.1)	31 (12.9)	25 (10.1)	81 (11.0)
	Never undergone HRT	178 (71.8)	172 (71.4)	169 (68.1)	519 (70.4)
**Knowledge of menopause**					
	Not knowledgeable at all	6 (2.4)	2 (0.8)	1 (0.4)	9 (1.2)
	Slightly knowledgeable	43 (17.3)	44 (18.3)	48 (19.4)	135 (18.3)
	Moderately knowledgeable	134 (54.0)	144 (59.8)	141 (56.9)	419 (56.9)
	Very knowledgeable	59 (23.8)	44 (18.3)	47 (19.0)	150 (20.4)
	Extremely knowledgeable	6 (2.4)	7 (2.9)	11 (4.4)	24 (3.3)
**Social media engagement**					
	Never	6 (2.4)	2 (0.8)	12 (4.8)	20 (2.7)
	Less than weekly	13 (5.2)	13 (5.4)	7 (2.8)	33 (4.5)
	Weekly	21 (8.5)	28 (11.6)	20 (8.1)	69 (9.4)
	Daily	109 (44.0)	100 (41.5)	109 (44.0)	318 (43.1)
	Several times a day	99 (39.9)	98 (40.7)	100 (40.3)	297 (40.3)

### Demographic Effects

Participants who reported being postmenopausal as opposed to perimenopausal were significantly less likely to feel worried about menopause (*b*=−0.630; SE 0.190; Wald *z*=−3.322; *P*<.001) controlling for all other demographic characteristics. Independent of menopause status, older participants were significantly less likely to report feeling worried (*b*=−0.492; SE 0.097; Wald *z*=−5.051; *P*<.001) or anxious (*b*=−0.463; SE 0.096; Wald *z*=−4.817; *P*<.001) about menopause, again controlling for all other demographic characteristics. Years of education and social media use did not have any statistically significant effects on affective responses. Higher social media use was associated with an increased likelihood to “like” a post (*b*=0.224; SE 0.069; Wald *z*=3.246; *P*=.001), save a post (*b*=0.254; SE 0.070; Wald *z*=3.614; *P*<.001), and comment on a post (*b*=0.251; SE 0.071; Wald *z*=3.561; *P*<.001). Years of education, age, and menopause status did not have a statistically significant effect on any expected behavioral responses to social media posts. A breakdown of participant demographics by condition can be found in Table 2.

**Table 2:** Demographic characteristics of UK perimenopausal and postmenopausal women in an online experiment on menopause-related social media narratives by experimental condition (N=737).

			Normal framing condition (n=248)		Distress framing condition (n=241)		Transformative framing condition (n=248)		Total
Age (y), mean (SD; range)			52.976 (8.455; 40-83)		53.386 (8.070; 40-75)		52.625 (8.487; 40-78)		52.992 (8.336; 40-83)
**Menopause stage, n (%)**									
	Perimenopause	130 (52.4)		117 (48.5)		138 (55.6)		385 (52.2)	
	Postmenopause	118 (47.6)		124 (51.5)		110 (44.4)		352 (47.8)	
Post-16 education (y), mean (SD; range)			5.516 (3.948; 0-20)		5.083 (3.602; 0-20)		5.149 (3.688; 0-18)		5.251 (3.750; 0-20)

### Effect of Experimental Condition on Affective Responses to Posts

Ordinal logistic regression models were used to examine how different framings of social media content about menopause influenced participants’ affective responses. Controlling for age, years of education, and frequency of social media use, the models showed that being exposed to posts depicting menopause as *distressing* significantly influenced participants’ reported emotional reactions. Specifically, compared to participants who viewed posts conveying menopause as *normal*, those who viewed *distress* posts were more likely to feel worried about menopause (β=.910; SE 0.170; Wald *z*=5.350; *P*<.001), less likely to feel reassured (β=−.970; SE 0.167; Wald *z*=−5.795; *P*<.001), more likely to feel confused (β=.818; SE 0.170; Wald *z*=4.820; *P*<.001), less likely to feel optimistic (β=−.708; SE 0.165; Wald *z*=−4.289; *P*<.001), more likely to feel anxious (β=.817; SE 0.168; Wald *z*=4.873; *P*<.001), less likely to feel empowered (β=−.540; SE 0.162; Wald *z*=−3.337; *P*<.001), and more likely to feel knowledgeable (β=.564; SE 0.163; Wald *z*=3.468; *P*<.001).

**Figure 2:**
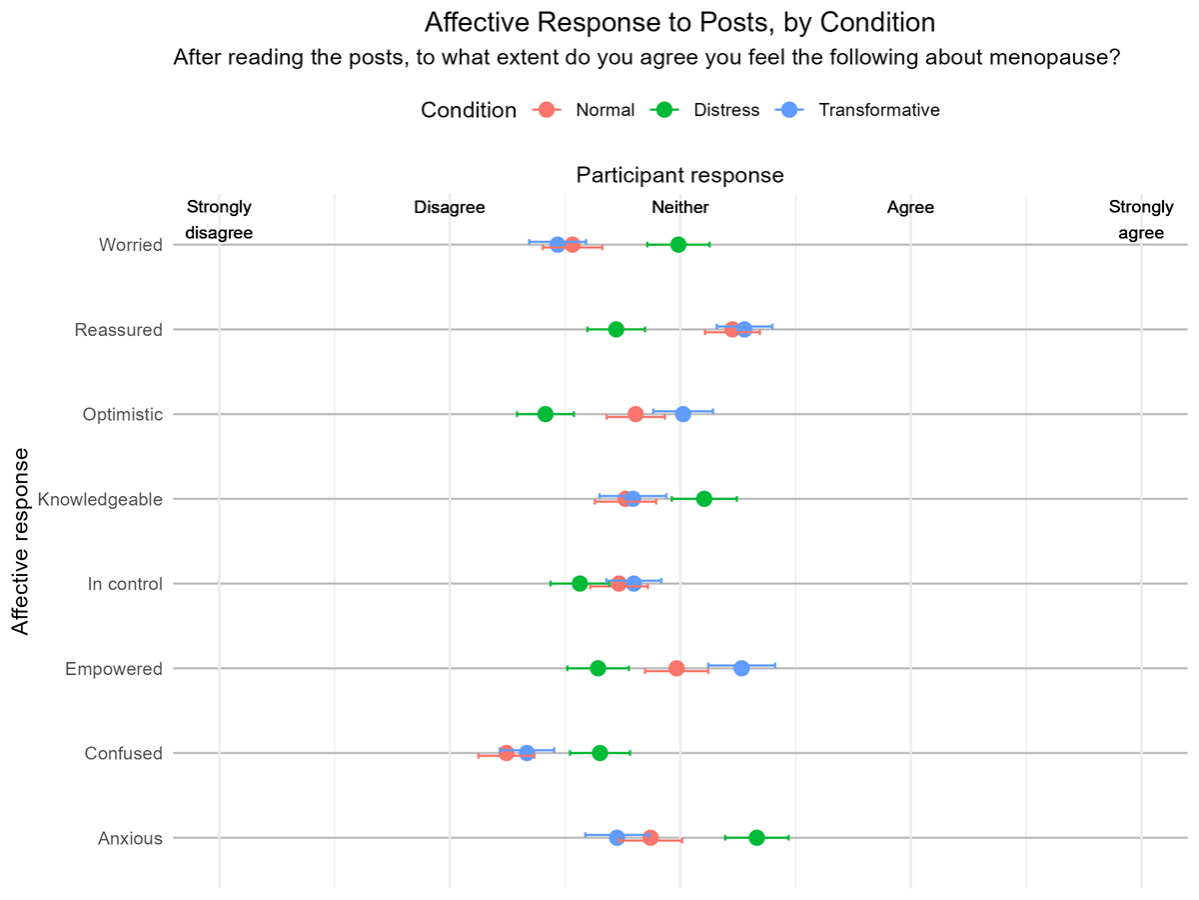
Affective responses to menopause-related social media posts among UK perimenopausal and postmenopausal women following exposure to the normal, distress, or transformative narrative conditions in an online experiment.

Being shown posts that conveyed menopause as a transformative experience did not have a statistically significant effect on affective responses compared to posts conveying menopause as a normal part of life (Figure 2).

### Effect of Experimental Condition on Expected Behavioral Responses to Posts

**Figure 3:**
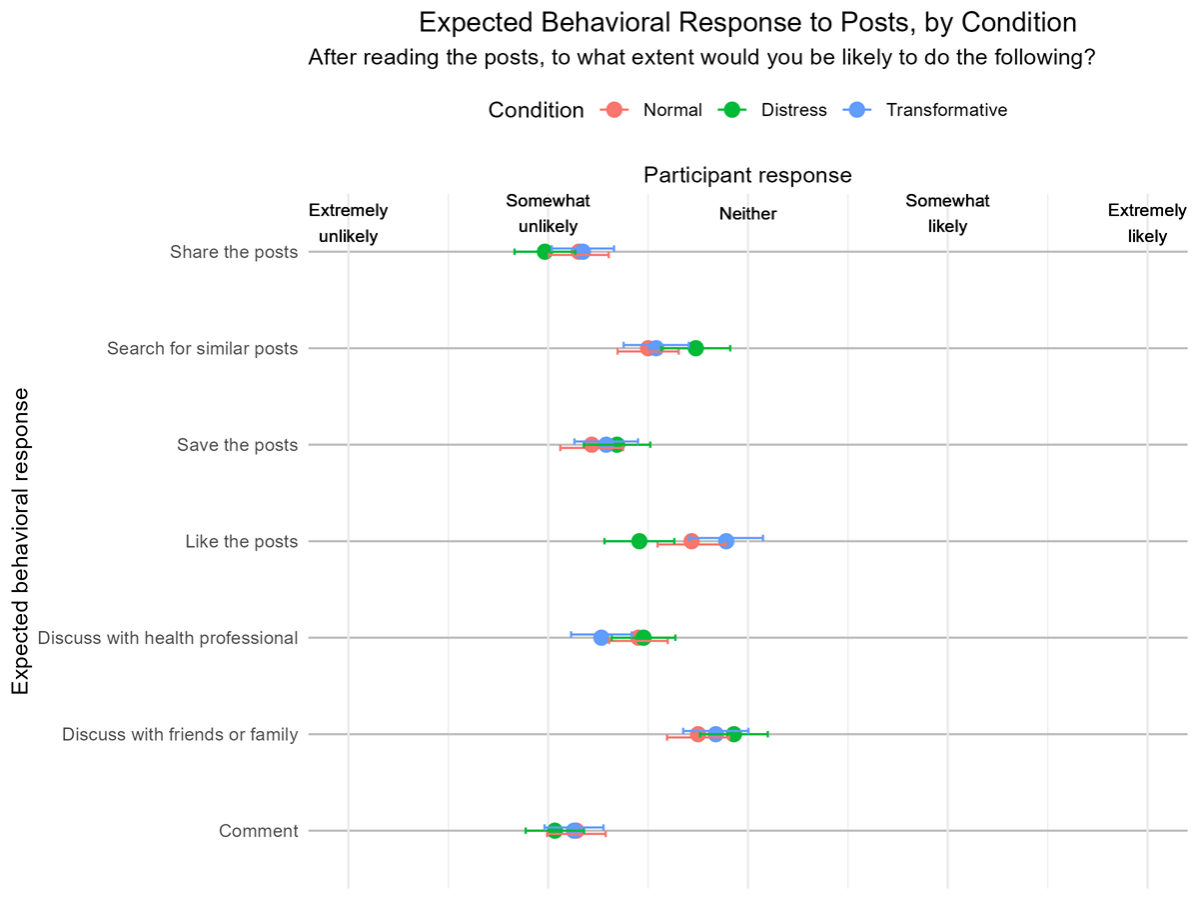
Expected behavioral responses to menopause-related social media posts among UK perimenopausal and postmenopausal women following exposure to the normal, distress, or transformative narrative conditions in an online experiment.

Neither the *distress* nor the *transformative* experimental conditions had statistically significant effects on any of the behavioral responses when compared to the *normal* reference category. This suggests that, compared to being shown posts conveying menopause as a normal part of life, exposure to posts portraying menopause as either distressing or a positive transformative experience did not meaningfully influence participants’ likelihood to like, share, save, comment on, search for, or discuss related content (Figure 3).

### Effect of Experimental Condition on Perception of Posts

**Figure 4:**
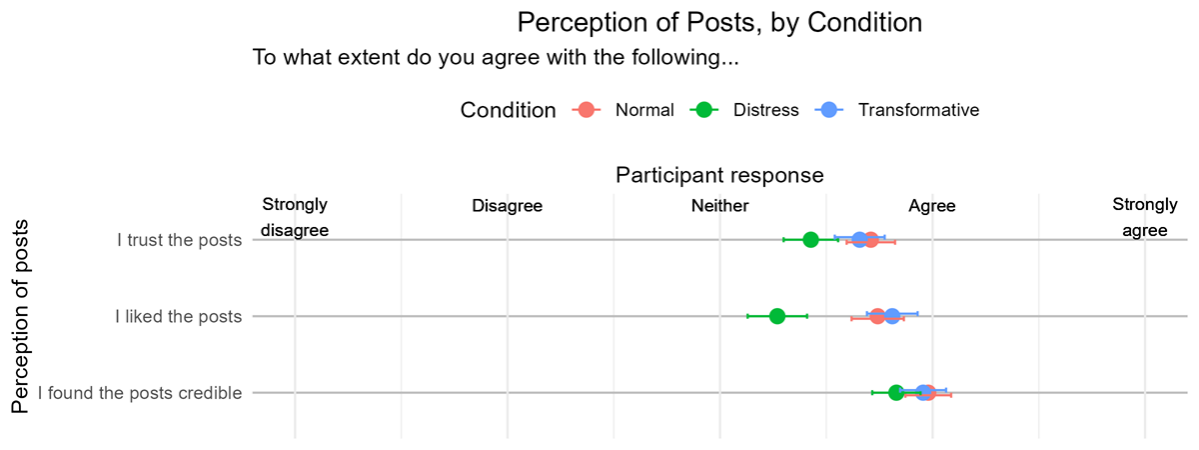
Perceptions of menopause-related social media posts among UK perimenopausal and postmenopausal women following exposure to the normal, distress, or transformative narrative conditions in an online experiment.

Experimental condition did not have an impact on participants’ initial perceptions of the posts, with the exception that those presented with posts depicting menopause as distressing were significantly less likely to report liking the posts they saw (*b*=−0.864; SE 0.171; Wald *z*=−5.068; *P*<.001; Figure 4).

**Figure 5:**
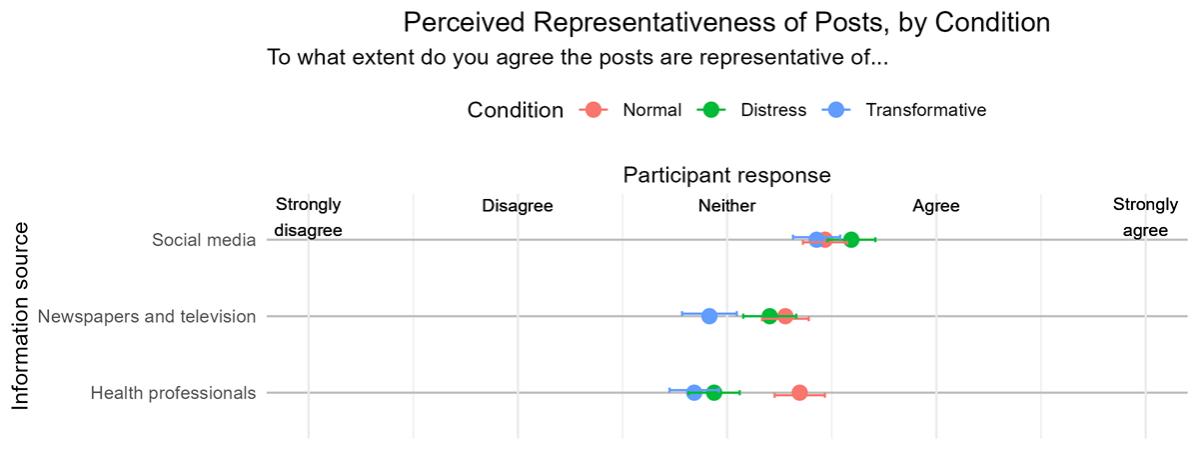
Perception of menopause-related social media posts as reflecting wider social media, traditional media, and health professional views among UK perimenopausal and postmenopausal women following exposure to the normal, distress, or transformative narrative conditions in an online experiment.

There were no statistically significant effects of experimental condition on the extent to which participants felt that the posts were representative of broader narratives found on social media. However, participants in the *transformative* condition were significantly less likely to agree that posts were representative of newspapers or television compared to those in the *normal* condition (*b*=−0.687; SE 0.165; Wald *z*=−4.152; *P*<.001). Participants in both the *distress* and *transformative* conditions were less likely to agree that the posts were representative of health professionals compared to those who saw *normal* messages (*distress* condition: *b*=−0.806, SE 0.168, Wald *z*=−4.808, and *P*<.001; *transformative* condition: *b*=−0.968, SE 0.167, Wald *z*=−5.802, and *P*<.001; Figure 5).

## Discussion

### Principal Findings

#### Overview

Our findings show that how menopause is framed on social media can meaningfully influence women’s immediate affective responses to online information. In an online experiment, participants who viewed posts portraying menopause as distressing reported greater levels of worry, confusion, and anxiety and lower levels of reassurance, optimism, and empowerment. Notably, shifts in affect did not extend to behavioral intentions to engage with the content (eg, liking or sharing posts). Below, we discuss these findings in relation to individual differences (such as menopause status and age), comparisons with existing literature, and practical considerations for online communication of menopause information. The discussion is organized around the 4 study objectives: the role of individual characteristics in shaping responses to menopause-related social media narratives, the impact of narrative condition on affective responses, the impact of narrative condition on expected behavioral responses, and participants’ perceptions of the posts and their representativeness of wider menopause narratives.

#### Individual Characteristics: Menopause Stage and Age Each Independently Influenced Worry and Anxiety

Participants who were postmenopausal reported significantly less worry about menopause, and older participants were less likely to feel worried or anxious even when controlling for menopause status. This finding suggests that age and menopausal stage make separate contributions to affective responses rather than one fully accounting for the other. It aligns with findings of prior research that has shown that postmenopausal women typically hold more positive attitudes toward menopause than premenopausal women [21,33]. Two-thirds of postmenopausal women report feeling better or at least neutral about menopause after the transition compared with before [34]. Perimenopausal women, in contrast, are more active information seekers online, being twice as likely to search on social media than postmenopausal women [21]. With lived experience to draw on, postmenopausal women may be able to reflect on and contextualize existing narratives about menopause, recognizing nuances that speak to the individual nature of “local biologies” [35] through which expectations, knowledge, and psychosocial factors shape experience. Evidence from life span research similarly indicates that older adults typically report less frequent worry, including about health [36]. Together, these findings underscore the value of earlier, credible information to allay anticipatory anxiety and support women who have not yet begun menopause. Although many women delay information seeking until the onset of perimenopausal symptoms [37], providing accessible guidance earlier in the life course, for example, in schools and at routine health checks and appointments [38], and adopting a more inclusive approach to menopause support and education [39] may help younger and perimenopausal women calibrate expectations and feel better equipped to navigate the transition.

#### Affective Responses: Exposure to Distress-Framed Posts Increased Negative Emotional Responses

A key finding of this study is that framing social media posts about menopause in distressing terms increased negative affect. Compared with participants who viewed posts that described menopause as a normal experience, those exposed to distress-framed posts reported higher worry, confusion, and anxiety and lower reassurance, optimism, and empowerment. These effects remained after controlling for age, educational level, and social media use. This pattern suggests that distress-framed narratives of menopause might shift affect in the moment, potentially narrowing perceived coping resources and elevating negative emotion. In contrast, positioning menopause as a normal life stage appeared to create a more positive affective response to social media posts. This is consistent with work showing that understanding what to expect can shape appraisals of menopausal experiences [40] and that better knowledge is associated with more positive experiences [41]. Digital spaces have been shown to cultivate affective atmospheres that modulate user experience [42]. Related research on emotional contagion shows that the emotional tone circulating in social feeds can shift users’ subsequent emotions and expressions, underscoring how local online climates matter in terms of how health narratives feel in the moment [43]. However, shifts in how content makes people feel do not necessarily translate into transmission behaviors such as liking or sharing, particularly when typical engagement cues are absent.

#### Behavioral Responses: Experimental Condition Did Not Influence Intended Behavioral Responses

Framing menopause as distressing or transformative had no impact on intended behaviors toward online information compared with framing it as a normal part of life. Despite strong effects on affect, neither distress nor transformative (vs normal) framing significantly changed intentions to like, share, save, comment, search, or discuss a post. Overall, reported behavioral intentions to engage with the social media posts were low across all narrative conditions. Retransmission rates for health content are often modest relative to other content types [44]. Many people obtain what they need from online health sources without visible participation, a pattern of passive use often described as “lurking” [45]. For menopause in particular, information seeking frequently serves as sense making and validation rather than public sharing [23,46]. Source and platform cues also shape willingness to act. Behavioral intentions vary with the identity of the source [47], and trust judgments about the source predict intentions to act on health advice [48-50]. Tweets from health organizations are more likely to be retransmitted than those from non–health organizations [44]. Social signals can function as bandwagon cues, where popularity is read as credibility [51], and these cues interact with message features to influence perceived credibility [52]. Our stimuli intentionally removed source identity and social proof, which likely dampened the reported expected behavioral intentions. Finally, translating online material into conversations with others is not always easy to achieve. For example, people are often reluctant to disclose to health care professionals that relevant health information came from the internet, sometimes attributing it to family or friends [53], and online inputs may be incorporated into health decision-making in more indirect, nuanced ways [54].

In addition to studying engagement behaviors such as sharing and commenting, future research should investigate how narrative framings of social media messages influence health-related behavioral responses to menopause information. Meta-analytic work on fear appeals shows that effective behavior change is more likely when messages that elicit fear or alarm are accompanied by clear information about how best to respond [55,56]. Messages signaling “what to do” in response to information about menopause often include high-agency tasks such as seeking HRT, booking general practitioner appointments, or purchasing recommended services and products. In the United Kingdom, recommended actions have also been amplified by celebrity-led menopause narratives, alongside reported increases in demand for HRT and ongoing debate about medicalization [57,58]. However, these routes are structurally constrained: access to general practice remains difficult and uneven, with persistent dissatisfaction about obtaining appointments despite record numbers of appointments being delivered in general practice overall [59]. The time, effort, and costs of navigating care constitute a burden on individuals that can lead to intervention-generated inequalities when solutions rely heavily on individual agency [60,61]. It is important that researchers and public health communicators closely consider the “agentic demands” placed on recipients of health information [62] and foreground feasible options alongside broader structural support so that widening online discourse about menopause does not center on solutions that many cannot enact.

#### Perceptions of Posts: Distress-Framed Posts Increased Perceived Knowledge but Reduced Post Likability

Although participants were less likely to report liking distress-framed posts about menopause, they reported feeling more knowledgeable about menopause after viewing them compared to posts conveying menopause as a normal stage of life. A possible explanation for this is that it is consistent with established negativity effects, where negative or threatening information is treated as more informative even if it is viewed as undesirable or unpleasant [63]. People preferentially attend to and remember threat-related material. In response to a given stimulus, negative emotions can enhance memory and be judged as more informative in certain circumstances compared to stimuli eliciting a more positive emotive response [64]. Emotional arousal accounts suggest that arousal sharpens competition for attention and memory, giving negative, high-priority cues an advantage for memory and processing of information. This can boost a sense of learning despite producing negative affect associated with the stimuli [64,65].

#### Perceived Representativeness: Distress- and Transformative-Framed Posts Were Viewed as Less Representative of Health Professionals

Compared to the *normal* condition, both distress and transformative posts were seen as less representative of health professionals, and transformative posts were also seen as less representative of newspapers or television. The fact that distress narratives were seen as less representative of health care professionals resonates with recent studies suggesting that women are not always taken seriously by health professionals when presenting with severe menopause symptoms [66]. Recent media attention regarding celebrities’ menopause experiences has opened up discussions on menopause, particularly regarding symptoms and HRT, although there have been suggestions that these well-publicized negative accounts contribute to the medicalization of what is usually a natural process [67]. Empowerment-based narratives emphasize the importance of incorporating both medical and psychological dimensions into menopause management [7], with some arguing that menopause should also be seen as an opportunity for growth, self-realization, and transformation [68]. However, many women report feeling overlooked or invisible during menopause [69], and the absence of relatable experiences can foster a sense of isolation and invisibility [67]. That transformative posts were seen as less representative of television or media more broadly perhaps reflects a cultural expectation in which Western cultures have tended to align menopause with aging and fertility, whereas in other cultures where menopause is viewed more positively, symptoms are expressed less frequently [70] and messages on transformation and growth are more prominent [71].

Encouraging a diverse expression of menopause narratives online can better reflect the highly individual nature of the transition, resonate with a wider range of lived experiences, and help reduce stigma and open discussion [22,72]. However, our findings also suggest a need to be mindful of the affective costs of distress framings. This is not an argument for curating or suppressing accounts of menopause distress but for acknowledging well-documented negativity effects, where people often treat negative information as more informative and remember it more strongly [63,64]. In convoluted online environments, negatively framed content can also be privileged by diffusion dynamics: moral-emotional and negative language tends to travel further and faster on social platforms, increasing visibility relative to neutral or positive narratives [73,74]. Recent qualitative research on perimenopause and menopause messages received as anticipatory education found that participants tended to judge memorable messages as negative or ambivalent, suggesting cognitive biases favoring negative menopause information [75]. Additionally, participation inequality on social media means that many users consume but rarely post, potentially skewing what appears to be representative of “ordinary” experiences [76]. Foregrounding distress-framed narratives of menopause may increase overall concerns, but only framing the transition as “normal” or “natural” risks dismissing women with severe and debilitating symptoms or who experience premature or surgically induced menopause [77]. The uniqueness of menopause and the fact that women may adopt multiple normal, transformative, or distressing narratives to describe their experiences [22] suggests a need for more nuanced menopause narratives across both online and traditional media.

### Limitations

Posts were anonymized, redacted, and standardized, and creator and platform cues were removed to reduce confounds. This removed the potential influence of features of social media posts such as the profile of the creator, current number of likes and interactions, or platform preferences. However, this also reduced the ecological validity of the stimuli because source identity and bandwagon cues often shape perceptions of credibility and willingness to engage with social media feeds [52]. Furthermore, behavioral outcomes were self-reported intentions rather than observed behaviors. Future work could incorporate trace data or follow-ups to capture enacted behaviors in response to viewing different social media narratives about menopause. Finally, our measures captured immediate reactions following a brief, controlled exposure to social media posts. More longitudinal designs are needed to test whether narrative-driven affective shifts accumulate or dissipate over time and potentially influence broader experiences and beliefs about menopause.

### Conclusions

This study shows that how menopause is framed on social media influences perceptions and immediate affective responses to online health content. In an online experiment, distress-framed posts increased worry, confusion, and anxiety and reduced reassurance, optimism, and empowerment. Crucially, neither distress nor transformative narratives altered behavioral intentions to like, share, save, comment on, search for, or discuss menopause posts. Overall, narrative tone shaped affective responses without shifting intended behaviors. Encouraging diverse menopause narratives can better reflect the breadth of women’s experiences, reduce stigma, and promote open conversation. At the same time, negative framings may become disproportionately salient on social media, potentially distorting perceptions and beliefs. Future work should examine whether such framing effects endure, how they interact with source and platform cues, and whether more balanced narratives improve women’s everyday experiences of menopause.

## Data Availability

The datasets generated or analyzed during this study are available in the Open Science Framework repository [32].

## Abbreviations

HRT: hormone replacement therapy
